# Combination of Multidimensional Instrumental Analysis and the Ames Test for the Toxicological Evaluation of Mineral Oil Aromatic Hydrocarbons

**DOI:** 10.1021/acs.jafc.2c05970

**Published:** 2022-12-16

**Authors:** Andrea Hochegger, Reinhard Wagenhofer, Sanja Savić, Elisa Mayrhofer, Michael Washüttl, Erich Leitner

**Affiliations:** † Institute of Analytical Chemistry and Food Chemistry, University of Technology Graz, Stremayrgasse 9/II, 8010 Graz, Austria; ‡ Department for Microbiology and Cell Culture, Austrian Research Institute for Chemistry and Technology, Franz-Grill-Straße 5, Objekt 213, 1030 Vienna, Austria

**Keywords:** MOAH, HPLC-GC-FID, GC × GC-ToF, Ames assay, miniaturized Ames assay

## Abstract

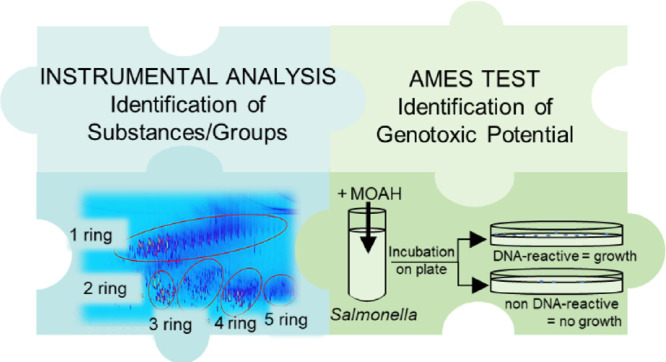

Mineral oil aromatic hydrocarbons (MOAHs) include mutagenic and carcinogenic substances and are considered a potential health risk. Current methods address the total MOAH content but cannot address the actual toxicological hazard of individual components. This work presents a combined methodology closing those gaps: high-performance liquid chromatography (HPLC) coupled to gas chromatography with flame ionization detection was used to determine the MOAH content. To characterize present substance classes, comprehensive two-dimensional gas chromatography with time-of-flight mass spectrometry was applied. Preparative HPLC separated MOAHs into subgroups, which were tested with a miniaturized Ames test evaluating DNA reactivity of isolated fractions. Combining these methods allowed a correlation between present subgroups and DNA reactivity. The developed approach was applied to a mineral oil and distinguished between not DNA-reactive mono- and diaromatics and DNA-reactive tri- and polyaromatics, providing a proof of concept. Hereinafter, it will be applied to diverse sample matrices including mineral oils, food, and food contact materials.

## Introduction

In 2019, the European Food Safety Authority published a rapid risk assessment of mineral oil aromatic hydrocarbons (MOAHs) in infant food, dealing with the findings of a foodwatch study, in which 8 of 16 samples showed an MOAH contamination ranging from 0.5 to 3.0 mg/kg.^1,2^ Taking into account its own scientific opinion already published on this issue in 2012, those findings were considered to be of concern for human health.^1,3^ Findings were also reported by several other authors in many different kinds of food matrices and in varying concentrations,^4−6^ further sparking the debate.^7−9^ Only recently, the Standing Committee on Plants, Animals, Food and Feed published a new statement on MOAHs in food, in which they agreed to withdraw or recall products from the market, if their MOAH content is above a defined limit of quantification.^10^

From an analytical point of view, MOAHs are–together with mineral oil saturated hydrocarbons (MOSHs)–part of mineral oil hydrocarbons (MOHs). MOHs are complex mixtures of hydrocarbons originating from petroleum and petroleum products.^11^ They end up in food mainly via three routes: First, because of their allowed use as highly cleaned products in several applications, e.g., microcrystalline wax (E905) as a surface treatment for fruits and vegetables, chewing gum, or confectionary, according to regulation (EC) No. 1333/2008 on food additives, with no defined maximum amount (“at quantum satis”),^12^ second, because of the transfer from a food contact material, and third, as a contamination with unknown composition and origin because of their ubiquitous presence in our ever day life (e.g. contamination from combustion motors, particular matter, lubricants from production machines, ...).^3,8,11^ MOSHs consist of branched and unbranched open chain hydrocarbons and cyclic hydrocarbons, and their bioaccumulation in several human tissues is considered to be a potential concern.^3,11^ The MOAH fraction makes up 15–30% of the total MOH and consists of aromatic substances having up to seven rings, with a different degree of alkylation. This fraction is considered to be potentially mutagenic and carcinogenic.^1,3,11^ Depending on the ring numbers and the degree of alkylation, MOAH compounds may act as DNA-reactive metabolites and tumor promoters or are carcinogenic through a nongenotoxic mechanism. While it is assumed that mono- and diaromatics and highly alkylated compounds are not metabolized to DNA-reactive metabolites, the mutagenic and carcinogenic potential of the MOAH fraction was previously related to the presence of 3–7 ring polycyclic aromatic compounds (PACs).^3,13,14^

Due to their complexity, risk assessment of the mixture of MOAHs is difficult. Even though the fraction consists of substances or substance classes with different modes of action some of which might even be nonrelevant, a worst-case assumption needs to be applied for all unknown components, which have to be categorized as DNA-reactive. For these compounds, the threshold of toxicological concern foresees a generic limit of 0.15 μg/day for a 60 kg person. A 120-fold higher limit would be applicable, however, only if any concerns for DNA-reactivity could be excluded.^3,8,13−15^ Only if substances were to be identified by routinely performed and detailed characterization, a substance-specific risk assessment would be possible to evaluate the actual toxicological concern.^8^

For this reason, the analysis of MOSH and MOAH has gained prominence over the past few years. Various authors have already discussed the existing challenges, resulting mainly from the lack of standardized and validated methods for different food matrices.^16−19^ Sample preparation needs to be done on the basis of a very high adjustment to the matrix “food”. Thereby, saponification to remove fat, aluminum oxide clean-up of the MOSH fraction, and epoxidation of the MOAH are only some of the crucial steps necessary.^8,19−22^ State-of-the-art analysis is done using high-performance liquid chromatography coupled online to gas chromatography with flame ionization detection (HPLC-GC-FID).^22,8^ The two fractions MOSH and MOAH are a result of the analytical separation using normal-phase liquid chromatography (LC) with a silica gel column and gas chromatography with flame ionization detection (GC-FID) for quantification.^11,23−25^ This determines the unresolved complex mixtures (UCM) of MOSH and MOAH only as a sum parameter, usually without further detailed interpretation of their origin or the composition of the fractions. However, the information of what is beneath the UCM is very much needed for the risk assessment and hazard characterization of the contamination that is present, in particular for the potentially mutagenic and carcinogenic MOAH. For this problem, two-dimensional comprehensive gas chromatography (GC × GC) is the method of choice which can determine, if three- and poly-ring aromatics are present.^1,5,8^ Recently, published methods deal with the routine identification and quantification of these sub-groups.^26,27^ Koch et al. presented a ring-specific separation of MOAHs using donor-acceptor complex chromatography. HPLC-GC-FID was used to quantify the separated fractions and GC × GC with a time-of-flight mass spectrometer (ToF-MS) to characterize them.^26^ Bauwens and co-workers proposed a fully integrated HPLC-GC × GC-ToF-MS/FID system, combining the traditional one-dimensional HPLC-GC-FID analysis with the two-dimensional approach, including MS characterization and FID quantification of sub-classes.^27^ It was demonstrated by the authors that these methods are capable of providing the much-needed information on detailed MOAH characterization and quantification of potentially relevant subgroups. The systems and methodologies used, however, are complex, and their use on a daily basis for many different samples is a questionable issue. Furthermore, the relevance for human health remains unclear.^8,14^

To evaluate the toxicological relevance of mineral oils, several alternative methods such as the mouse skin painting assay or the IP346 were previously proposed.^14,28,29^ In a different manner to analytical approaches, neither of these methods relies on the identification of critical substances, but instead they evaluate critical effects based on the whole mixture.

Among these probably the most famous is the classical mouse skin painting assay where mineral oil samples are repeatedly applied to mouse skin to determine their dermal carcinogenicity.^30^ The drawbacks here have been discussed previously in the literature^7^ including, time, as treatment periods of at least 78 weeks are usually applied, and also animal welfare issues, with additional strong legislative pressure for a shift to animal free testing.^31,32^

To simplify testing, the IP346 method was readily developed which determines the weight percentage of PACs in dimethyl sulfoxide (DMSO) extracts of lubricant base oils.^28,33,34^ Levels of ≥3% of PAC correlate well with tumor formation in the mouse skin painting assay.^28,29^ However, this procedure has two main disadvantages. On the one hand, the method is only applicable to lubricant oil samples with strict exclusion of samples of different origin and oils with additives. Furthermore, the required sample quantities are immense, and obtaining these from real food samples is simply unrealtistic.^29^

Another alternative widely applied for the testing of mineral oils is a modified version of the classical Ames test, using histidine auxotrophic *Salmonella* strains to score DNA-reactive effects upon sample-induced reestablishment of histidine biosynthesis.^35−38^ Compared to the IP 346 method, the modified Ames test was shown to be superior in the prediction of mutagenic effects originating from mineral oils and shows a good correlation to mouse skin painting assay results.^28^ Furthermore, the Ames test has high relevance to predict carcinogenicity, as about 80% of Ames positive substances indeed induce carcinogenic effects in rodents,^39^ which is quite high given the intrinsic variability of the assay (80–84% inter-laboratory repeatability).^40^ Even though the test cannot detect all DNA-reactive, mutagenic compounds at levels of 0.15 μg/day, a comparison of several genotoxicity bioassays regarding their ability to detect trace amounts of mutagens in complex mixtures identified the assay as the most sensitive method.^41^ The test is performed on agar plates, although once again the method suffers from the high sample requirements. Different miniaturizations of the classical Ames test were previously developed to reduce the sample quantitites.^42^ The miniaturized Ames test used in this study has already been successfully applied to screen extracts of food contact materials^43^ and recycled polymers,^44^ i.e., complex mixtures like mineral oils, and benefits from higher sensitivity than the agar plate-based version.^45^

To evaluate the actual toxicological relevance of MOAHs present in food the current work aimed to combine the advantages of state-of-the-art analytical substance identification and biological assessment. Already existing and published methods were adapted and applied, such as silver silica chromatography to separate MOSHs and MOAHs,^31^ followed by donor–acceptor chromatography to isolate and enrich mono- and diaromatic and the tri- and polyaromatic compounds from the obtained MOAH fraction.^29^ The raw material and the separated fractions were comprehensively characterized using GC × GC-ToF, and isolated fractions were tested in the miniaturized Ames test. The data presented shows a proof-of-concept for the proposed analysis strategy using a commercially available mineral oil sample. This combined methodology will in future close the knowledge gaps in hazard characterization and risk assessment by allowing the testing of MOAH fractions in real food samples for their DNA-reactive potential.

## Materials and Methods

No unexpected safety hazards were encountered in this work.

### Experimental Overview

**Figure 1:**
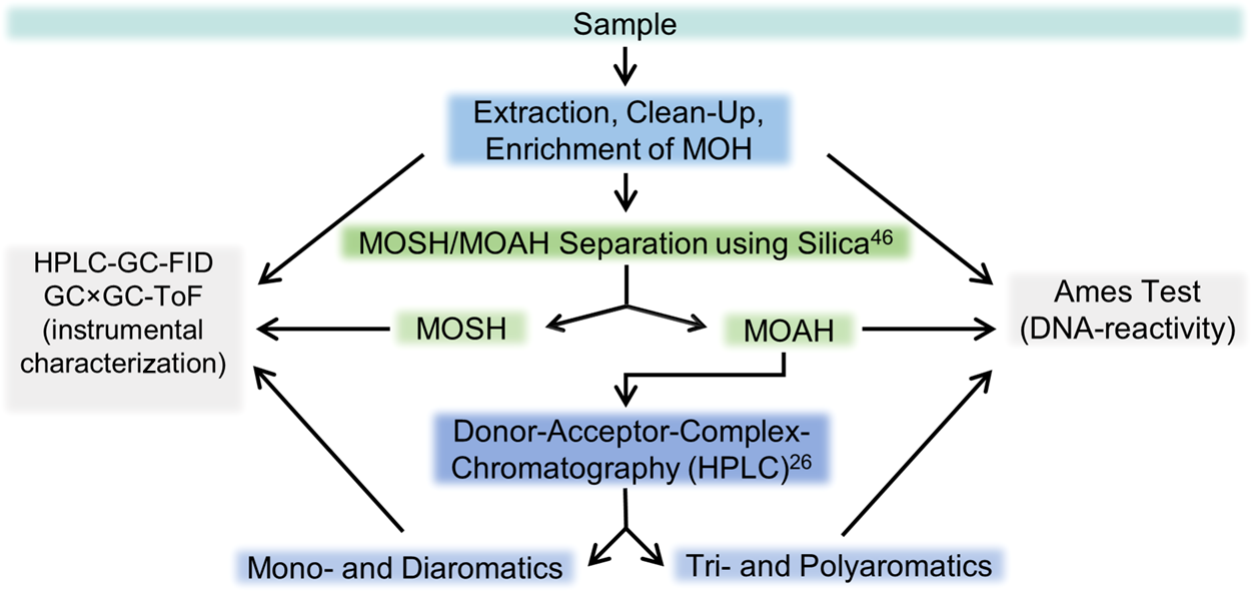
Scheme of the experimental setup.

Figure 1 gives an overview of the performed experimental procedure. In the first step, a mineral oil sample (MOLTOX Reference Oil No. 1, purchased at Trinova Biochem GmbH, Giessen, Germany) was analyzed for its mineral oil content, using state-of-art sample preparation techniques and HPLC-GC-FID for quantification. The present contamination was further characterized using GC × GC-ToF. Furthermore, an extract of the sample was directly evaluated in the miniaturized Ames test. Second, the MOSH and MOAH were separated from each other and individually tested using again instrumental analysis and the miniaturized Ames test. The (Ames positive) MOAH fraction was further separated into <3 ring aromatics and ≥ 3 ring aromatics, enriched, and tested again using the Ames test to identify the substance groups, being responsible for the positive result in the first test.

The procedure was applied to a mineral oil sample to do a proof of concept. The following sections describe the individual steps in detail.

### Determination of Mineral Oil (MOSH/MOAH) Content

The mineral oil sample was diluted to 200 mg/L in *n*-hexane (Picograde for Residue Analysis; LGC Promochem GmbH; Wesel, Germany) and an internal standard (MOSH/MOAH standard; Restek Corporation (Bellefonte, US) containing following substances in 1 mL ampules in toluene was added: *n*-undecane (C11; 300 μg/mL), *n*-tridecane (C13; 150 μg/mL), cyclohexylcyclohexane (CyCy; 300 μg/mL), cholestane (5-*alpha*-Cholestane; Chol; 600 μg/mL), 1-methylnaphthalene (1-MN; 300 μg/mL), 2-methylnaphthalene (2-MN; 300 μg/mL), *n*-pentylbenzene (5B; 300 μg/mL), perylene (Per; 600 μg/mL), and 1,3,5-tri-*tert*-butylbenzene (TBB; 300 μg/mL). Ten microliters were added to the sample to reach a final concentration of 1.5–6 μg/mL. The sample was analyzed for its MOSH/MOAH content using an online-coupled HPLC-GC-FID. For further characterization of the substance groups, present GC × GC-ToF-MS was applied as described in “instrumental analysis”.

Manual separation of MOSH and MOAH was done for 5 g of the mineral oil sample diluted with *n*-hexane as described by Fiselier et al.:^46^ a glass column was packed with 15 g 0.3% silver-nitrate on silica (29 g of activated silica gel 60 mixed with 1 g of 10% silver nitrate on silica, the latter pre-purchased at Sigma-Aldrich, St. Louis, USA). Elution of MOSH was done using 50 mL of *n*-hexane, the column was conditioned using 10 mL of hexane/dichloromethane 70:30 and MOAH was eluted using additional 80 mL of dichloromethane (HPLC grade, ≥99.8% CH_2_Cl_2_, CHEM-LAB, Zedelgem, Belgium). Both fractions were evaporated to dryness using a gentle stream of nitrogen. A clean separation was checked by HPLC-GC-FID and GC × GC-ToF using the same approach as for the pure oil described above in “determination of mineral oil content”.

### Separation of MOAHs

Mono- and diaromatic compounds were separated from tri- and polyaromatics using a method adapted from Koch et al.^26^ A Shimadzu LC-20 AD was used, equipped with a Nucleosil Chiral-2 5 μm column (250 × 4 mm, MACHEREY-NAGEL GmbH & Co. KG, Düren, Germany), a Prominence RF-20Axs fluorescence detector, and a fraction collector. Gradient elution was used at a flow rate of 1 mL/min. It started with 100% *n*-hexane (hold 3.2 min) and raised to 70% dichloromethane within 0.3 min (hold 6.8 min). The column was reconditioned to 100% *n*-hexane afterward (hold 9.7 min). Column temperature was set to 25 °C. Fractionation was controlled using the integrated fluorescence detector (Ex = 280 nm, Em = 380 nm). An injection volume of 10 μL of a 10 mg/L solution of separated MOAHs was used. The separated fractions were collected and evaporated to dryness. A clean separation was checked using HPLC-GC-FID and GC × GC-ToF using the same approach as for the pure oil described above in “determination of mineral oil content”.

### Instrumental Analysis

Quantitation of MOSHs and MOAHs was performed using an online-coupled HPLC-GC-FID system. The HPLC was a Shimadzu LC 20 AD model equipped with an Allure Silica 5 μm column (250 × 2.1 mm). The UV-detector was equipped with a D_2_-lamp set at 230 nm and 40 °C cell temperature. Gradient elution was used, starting with 100% *n*-hexane (flow rate 0.3 mL/min) and raised to 35% dichloromethane within 2 min (hold 4.2 min). The column was backflushed at 6.3 min with 100% dichloromethane (flow 0.5 mL/min; hold 9 min) and reconditioned to 100% *n*-hexane (flow rate 0.5 mL/min; hold 10 min). The flow rate was subsequently decreased to 0.3 mL/min until the next injection. The online-coupled GC was a Shimadzu GC 2030 dual-column and dual-FID system, equipped with two guard columns (Restek MXT Siltek (10 m × 0.53 mm id)) and two analysis columns (Restek MTX-1 (15 m × 0.25 mm id × 0.25 μm df)). The carrier gas was hydrogen with 150 kPa analysis pressure. The oven was programmed to 60 °C (hold 8 min), raised by 15 °C/min to 120 °C (hold 0 min), and followed by 25 °C/min to 370 °C (hold 6 min). The FID temperature was set to 390 °C. The “Chronect-LC-GC” software by Axel-Semrau was used to control the HPLC-GC interface. Data evaluation was performed using the software “LabSolutions” by Shimadzu Corporation for LC and GC data in version 5.92.

GC × GC analysis was performed using a PEGASUS BT 4D GC × GC-TOF-MS (LECO, St. Joseph, MI, USA), controlled by the Leco “ChromaTOF” software in version 5.51.50. The instrument consisted of a 7890B gas chromatograph (Agilent Technologies, Waldbronn, Germany) equipped with a cool on-column injector, an Agilent 7693A autosampler, a secondary internal oven, a quad-jet dual stage thermal modulator, and a time-of-flight mass spectrometer. The column configuration was a reversed polarity setup, with a 15 m × 0.25 mm i.d. × 0.25 μm Rxi-17Sil MS (Restek Corporation, Bellefonte, US) first dimension column connected via a SilTite μ-Union (Trajan Scientific and Medical, Victoria, Australia) to a 1.6 m × 0.18 mm i.d. × 0.18 μm ZB-1HT Inferno (Phenomenex, California, USA) second dimension column. These columns were temperature-programmed from 40 °C (hold 1 min) to 360 °C at 5 °C/min (hold 1 min) with a secondary oven offset of +15 °C. The modulator offset was also set to +15 °C. Helium was used as a carrier gas in constant flow mode (1.4 mL/min). Modulation time was 6 s. Spectra were collected in the *m/z* range from 50 to 700, with a scan rate of 100 spectra/s. The ion source was set to 250 °C, and the transfer-line was set to 340 °C. The detector voltage was relative to tune and was applied after the solvent delay of 240 s. Injection volumes were between 1 and 2 μL.

### Sample Preparation for Miniaturized Ames Assay

To prepare bioavailable samples for Ames testing, oil fractions were extracted with DMSO. DMSO extraction methods were adapted from the published literature.^35,37,38^ In short, 5–10 mg of the total MOAH fraction or the mono- and diaromatic and tri- and polyaromatic subfractions were added to 250 μL of DMSO, which was mixed vigorously for 1 min. The mixtures were incubated at 60 °C overnight and tested directly in the miniaturized Ames test the following day. For re-testing, samples were stored at −20 °C.

### Miniaturized Ames Assay

The Ames assay was performed based on the method’s supplier protocol Xenometrix.^47^ Exposure and reversion indicator medium were prepared according to ISO 11350:2012.^48^ In short, 10 μL of sample were applied in triplicate in 24-well plates, diluted 25-fold with exposure medium containing 5% (v/v) bacteria (*Salmonella typhimurium* TA98, approx. 10^9^ CFU/mL) and 4.5% (v/v) phenobarbital/β-naphthoflavone-activated rat liver S9, and incubated for 90 min at 37 °C under agitation. Subsequently, 2.6 mL of reversion indicator medium containing 0.2% (w/v) bromocresol purple indicator were added, and the content of each well was distributed into 48 wells of a 384-well plate. The plates were incubated for 48–72 h at 37 °C and scored by counting the yellow revertant wells in each 48-well unit. Samples were classified as positive if their mean revertant counts showed a ≥ 2-fold increase compared to baseline, the sum of the mean revertant count of the negative control plus one standard deviation.

## Results and Discussion

**Figure 2:**
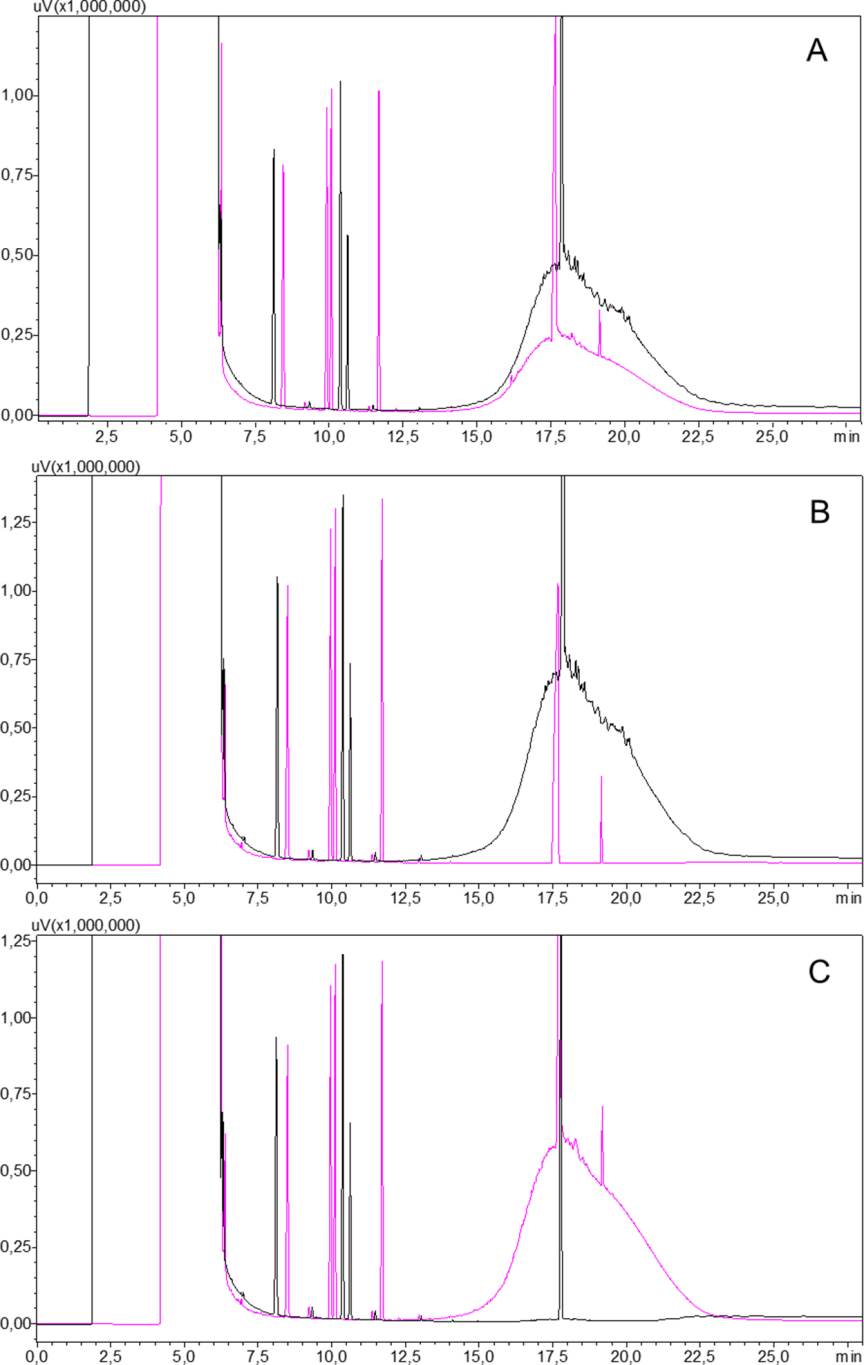
HPLC-GC-FID chromatograms of (A) total mineral oil sample (B) on silver silica separated MOSH fraction and (C) on silver silica-separated MOAH fraction. Black traces show MOSH, and pink traces show MOAH.

In the first step, the characterization of the mineral reference oil using online-coupled HPLC-GC-FID revealed that it consists of 70% MOSH and 30% MOAH (Figure 2A). A miniaturized Ames bioassay was done^35,37,49^ and gave a positive result, showing the DNA-reactivity of the mineral oil as such (data not shown). Since the oil is a complex mixture of thousands of chemicals, no conclusion about the substance or substance group triggering the positive response could be done due to the existing knowledge gaps.

Therefore, in the next step, the mineral oil was separated into its MOSH (Figure 2B) and MOAH (Figure 2C) fraction. Manual separation of 5 g oil using silver-silica resulted in 3.5 g MOSH and 1.5 g MOAH. Clean separation was confirmed by HPLC-GC-FID.

**Figure 3:**
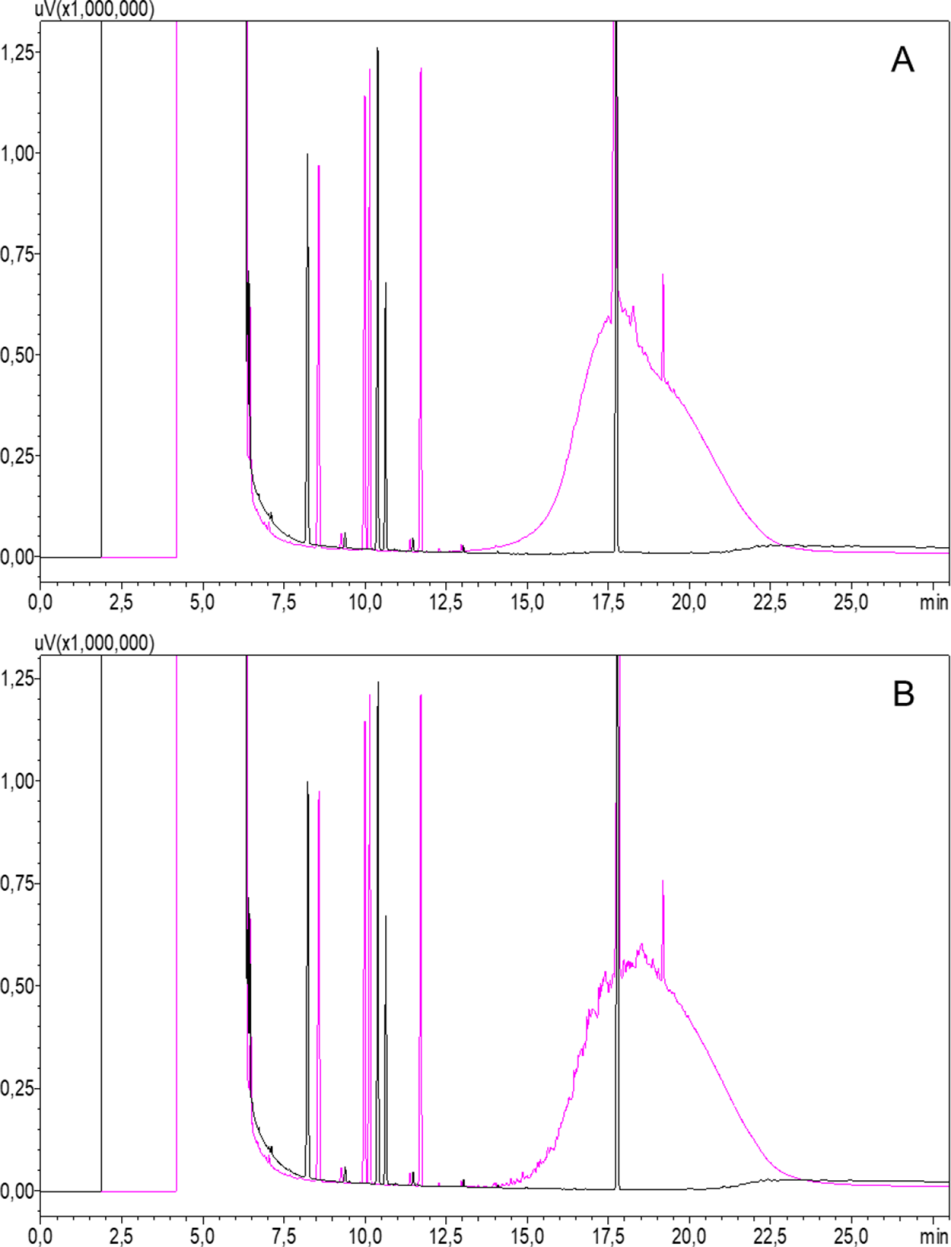
HPLC-GC-FID chromatogram of (A) isolated mono- and diaromatics and (B) isolated tri- and polyaromatics. Black traces show MOSH, and pink traces show MOAH.

The MOAH fraction was further separated into 1.1 g of mono- and diaromatics and 0.4 g of tri- and polyaromatics. At this point, it was no longer possible to see a significant difference between the isolated fractions in the HPLC-GC-FID chromatograms (Figure 3).

**Figure 4:**
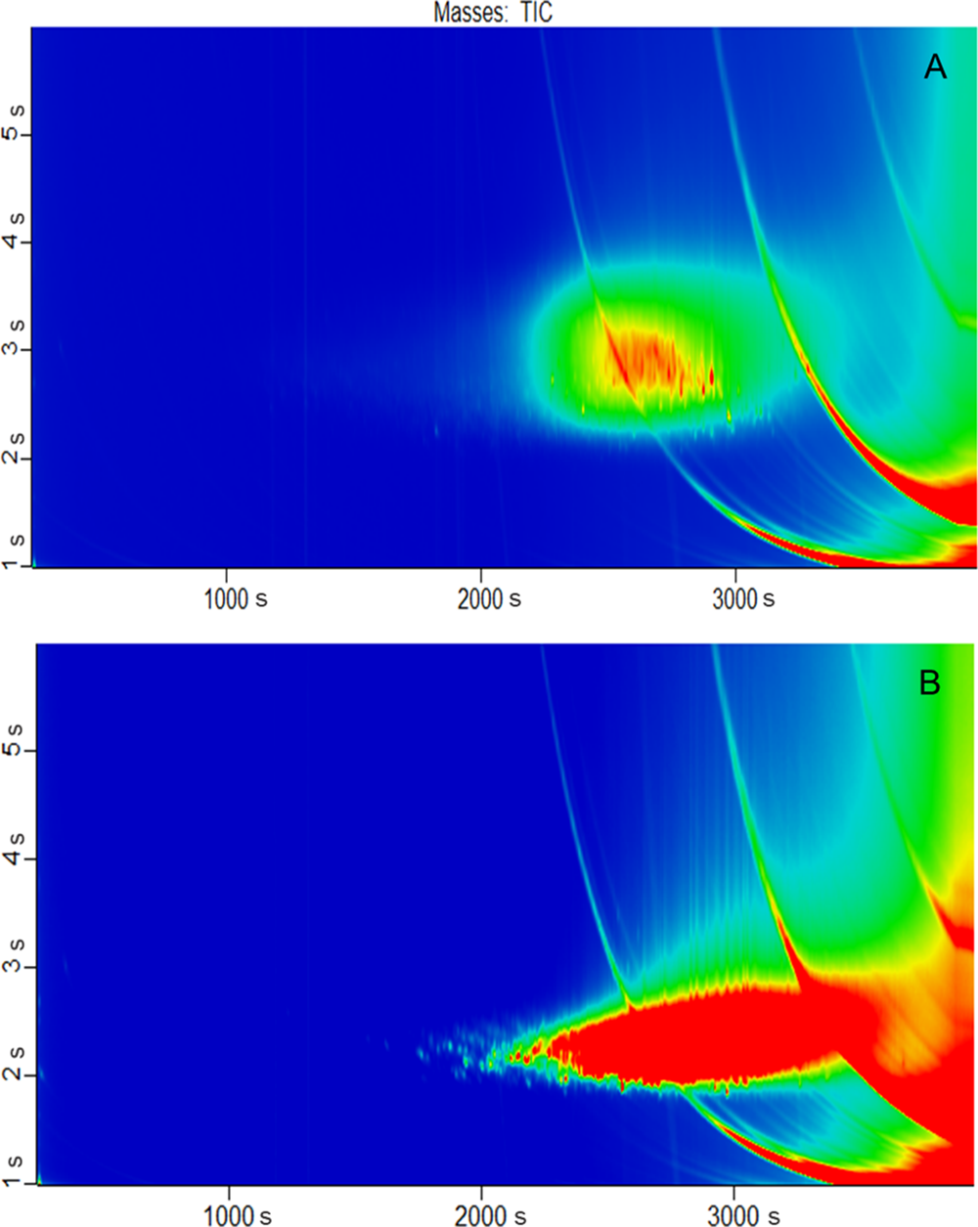
Total ion current of (A) isolated mono- and diaromatics and (B) isolated tri- and polyaromatics.

In comparison, the GC × GC-ToF gave much more information (Figure 4). It allowed for a detailed characterization of the present substances as initially proposed by Biedermann et al. and also described by several other authors, using filters and specific mass to charge ratios (*m/z*).^26,50,51^ For the mono- and diaromatic fraction, the identified substance classes included alkylated benzenes, indenes, naphthalenes, and biphenyls. In the tri- and polyaromatic fraction substance classes having 3 to 6 aromatic rings were identified, including those with heteroatoms (e.g. benzo[*b*]naphtho[2,1-d]thiophene and the respective isomers). It is also possible to determine the degree of alkylation to some extent. It ranged from no alkylation (PAH mother compound) to a high degree of alkylation. The monoaromatics had an alkyl chain length of up to C_25_, the indenes of up to C_13_, the naphthalenes of up to C_18_. The triaromatic compounds were represented by the nonalkylated phenanthrene and anthracene, but also by the alkylated variants up to C_13_-alkyls. Additionally, pyrene and fluoranthene were present, also their C_1_–C_5_ alkyl variants, as well as chrysene and benz[*a*]anthracene with alkyls up to C_13_.

Since with increasing ring number many more substances with the same molecular mass can be present, identification of single substances is becoming too complicated. Substances having 4.5 to 5 aromatic rings showed isomers up to C_14_-alkyls besides the nonalkylated mother compounds (*m/z* 152). For the 6 and 6.5 ring aromatics, alkylation up to C_4_ was observed in GC × GC at the very end of the chromatogram. Higher ring aromatics and higher alkylation may be present but could not be detected anymore with the used GC × GC method.

A lack of separation was seen for alkylated azulenes and dibenzothiophenes, which eluted in the tri- and polyaromatic fraction, although being two-ring aromatics. No clear identification was possible for alkylated acenaphthylenes, fluorenes, and dihydro phenanthrenes/anthracenes, since they all have the same *m/z*. Elution of those *m/z* was only seen in the tri- and polyaromatic fraction. However, it was proven with single substances that fluorene is eluting in the mono- and diaromatic fraction (data not shown). Furthermore, no other partly hydrogenated MOAH substances were identified, leading to the conclusion that the substances are alkylated acenaphthylenes.

**Figure 5:**
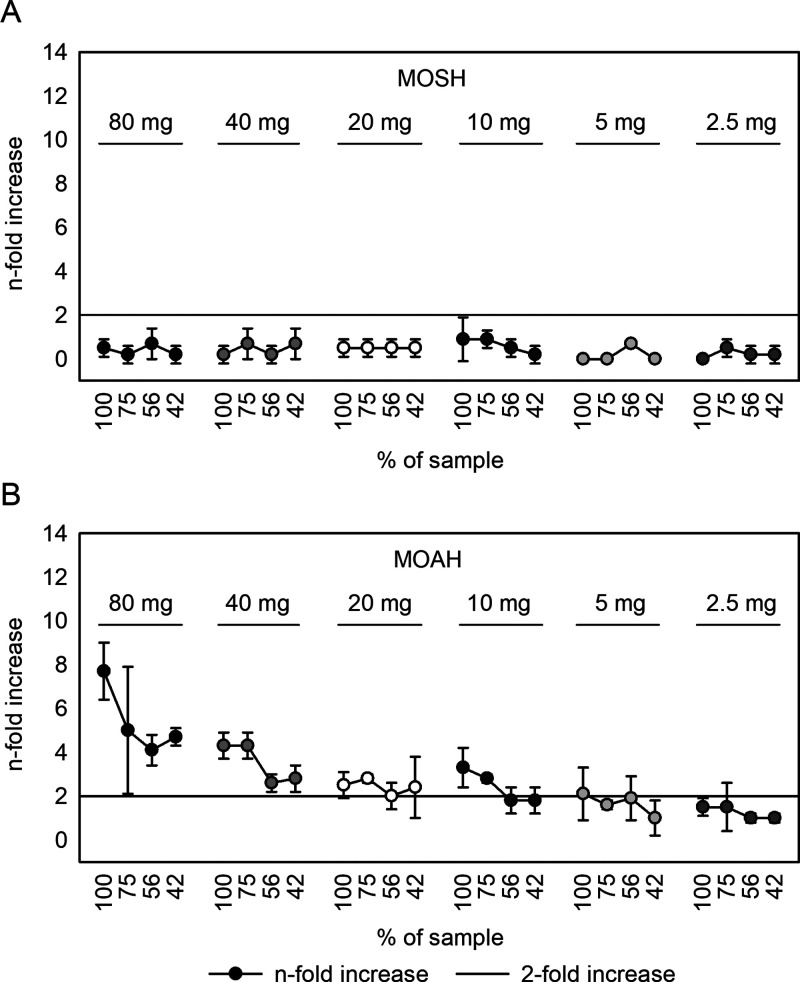
Miniaturized Ames analysis of DMSO extracts prepared from (A) total MOSH fraction and (B) total MOAH fraction of the mineral reference oil. Dilution series were prepared using different extraction ratios: 80–2.5 mg/250 μL DMSO.

To verify that the differences between the mono-and diaromatic as well as tri- and polyaromatic fractions in the GC × GC-ToF analysis translate into different DNA-reactive potentials, the isolated fractions were used to test DNA-reactivity in the miniaturized Ames assay. To account for the limited sample amount produced by chromatographic fractionation, the screening only focused on the *Salmonella typhimurium* strain TA98 in the presence of metabolizing enzymes (S9), as this proved to be especially sensitive for mineral oil testing throughout the literature.^49,35−38^ Bioavailable samples were prepared by DMSO extraction; however, procedures suggested in the literature had to be optimized to cope with limited sample availability.^36,14,29^ By continuous reduction of the sample quantity (80–2.5 mg/250 μL DMSO) and comparison of different heating variants (1 or 7 h at 60 °C, partially with regular mixing), stable signals could be generated already with 5–10 mg of total MOAH fraction (Figure 5B, Supplementary Figure 1). Furthermore, heating for at least 7 h at 60 °C in the drying cabinet yielded an additional gain of signal intensity (Supplementary Figure 1). Importantly, a comparative analysis of the MOSH fraction showed no DNA reactivity using the same extraction parameters (Figure 5A).

**Figure 6:**
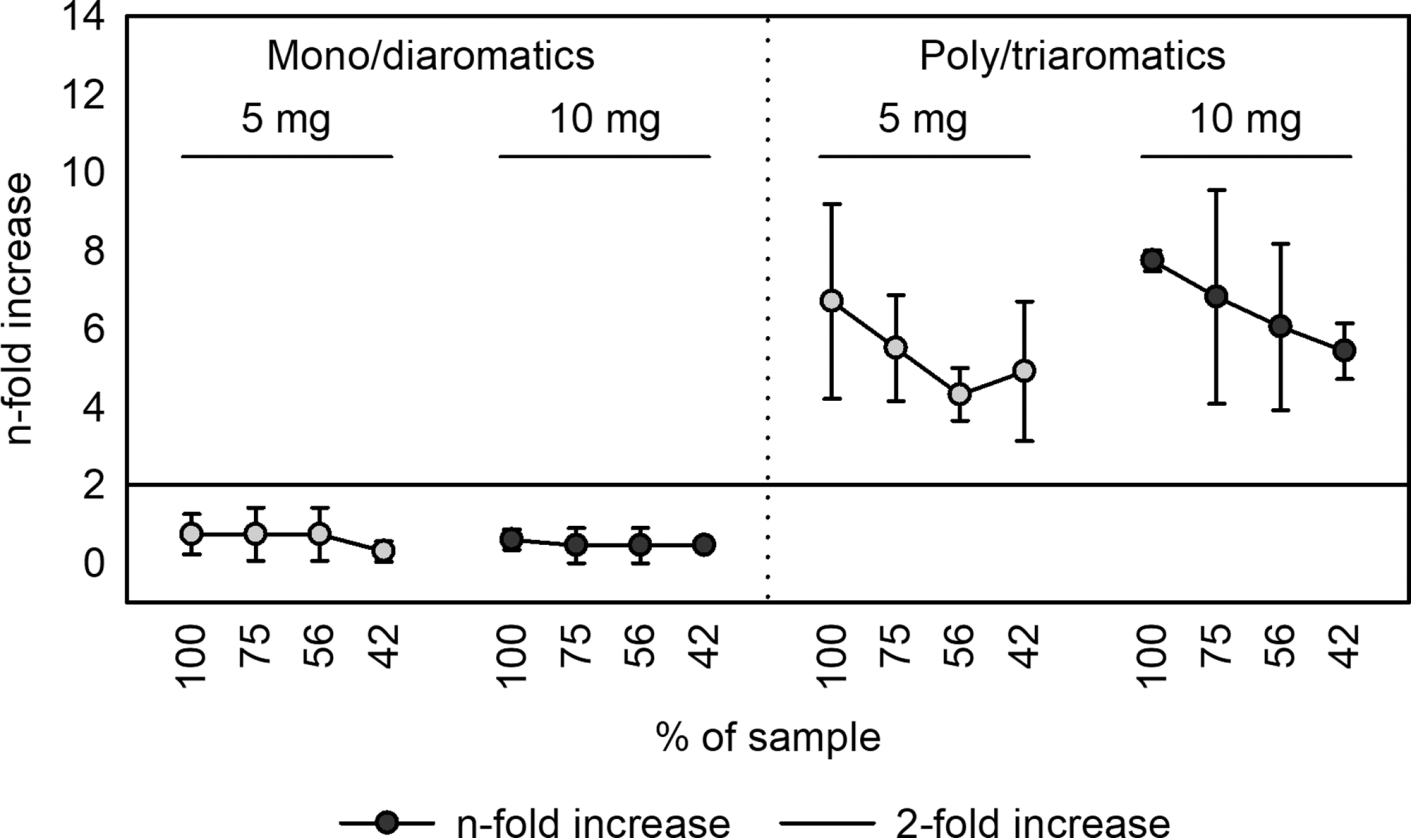
Miniaturized Ames analysis of DMSO extracts prepared form the mono- and diaromatic (left) and tri- and polyaromatic (right) subfraction of a mineral reference oil. Two extraction ratios, 5 mg/250 μL and 10 mg/250 μL DMSO, were compared.

Applying these adapted sample preparation conditions to test mono- and diaromatic and tri-, and polyaromatic MOAH subfractions of the reference mineral oil in the miniaturized Ames test showed that mono- and diaromatics compounds do not induce a detectable response in the assay, while tri- and polyaromatics clearly exhibit DNA-reactivity, an effect which was recovered at both tested concentrations (Figure 6).

In conclusion, a proof of concept was provided that a combination of instrumental analysis and Ames test can identify health relevant subfractions in the complex MOAH mixture of the tested mineral oil sample.

Previously published analytical methods for the analysis and the fractionation of mineral oils were successfully applied in an interdisciplinary workflow necessary to prepare defined mineral oil fractions for toxicological assessment. Furthermore, the Ames test has shown to have the potential to distinguish DNA-reactive from non-DNA-reactive MOAH subfractions in this setting. The adaptive measurements in sample preparation and testing conditions have proven successful compatibility with the chromatographic pre-work necessary for this kind of testing.

Although those results appear to be promising, there is much that still needs to be evaluated. First, little is known about the composition of other MOAH fractions. A profound risk assessment is best accomplishable with the knowledge of the components. This knowledge is provided by the GC × GC analysis, giving not only the ring number, but also the degree of alkylation. However, many factors are unclear for now: we saw a lack of separation for substances with heteroatoms and we also detected the individual PAH substances. Their influence on the Ames result needs to be evaluated and compared to other MOAH composition.

Furthermore, no conclusion can yet be drawn about the concentration relevant for human health. Further studies are needed to gain more information on this complex topic. Nevertheless, the proposed method provides an opportunity to fill gaps in the mineral oil risk assessment, leading to a more substance-based assessment, rather than considering the whole MOAH as DNA-reactive.

Future studies will deal with the characterization, isolation, and evaluation of MOAHs from different sources and of different compositions to generate the much-needed information for a more sophisticated understanding. A database shall be generated, including information on present substances and substance classes, their concentration, degree of alkylation, and the results of the Ames test. This may then allow for a more substance specific risk assessment, not necessarily considering the whole MOAH fraction as DNA-reactive. By generating this information, it may be possible in the future to predict health effects by analyzing only the amount and composition of MOAHs with analytical tools.

## Supporting Information Available

Supplementary Figure 1: Comparison of different DMSO extraction procedures of mineral oil for miniaturized Ames analysis (PDF)
