# Supplementary material for: Combination of Multidimensional Instrumental Analysis and the Ames Test for the Toxicological Evaluation of Mineral Oil Aromatic Hydrocarbons
Source: J Agric Food Chem. 2022 Dec 16;70(51):16401–9. doi: 10.1021/acs.jafc.2c05970 (PMC9801419; doi:10.1021/acs.jafc.2c05970)
Supplement: Supplementary file 1 — jf2c05970_si_001.pdf [file jf2c05970_si_001.pdf]

Supporting Information

for

Combination of Multidimensional Instrumental Analysis and the Ames Test for the Toxicological Evaluation of Mineral Oil Aromatic Hydrocarbons

Andrea Hochegger[1*], Reinhard Wagenhofer[1], Sanja Savić[2], Elisa Mayrhofer[2], Michael Washüttl[2], Erich Leitner[1]

[1]University of Technology Graz, Institute of Analytical Chemistry and Food Chemistry, Stremayrgasse 9/II, 8010 Graz, Austria

[2] Austrian Research Institute for Chemistry and Technology, Department for Microbiology and Cell Culture, Franz-Grill-Straße 5, Objekt 213, 1030 Vienna, Austria

**Corresponding author:*

andrea.hochegger@tugraz.at,

+43 316 873 32552

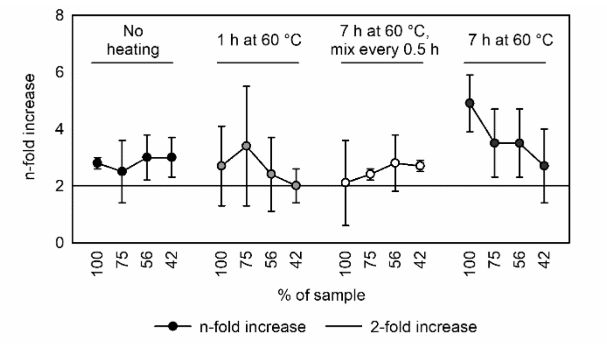

Supplementary Figure 1: Miniaturized Ames analysis of DMSO extracts prepared from the total MOAH fraction of a mineral reference oil. Different extraction procedures were compared: no heating treatment, heating for 1 h at 60 °C, heating for 7 h at 60 °C with mixing every 30 min, heating for 7 h at 60 °C (from left to right).
